# Patient safety improvement with the patient engagement in Iran: A best practice implementation project

**DOI:** 10.1371/journal.pone.0267823

**Published:** 2022-05-11

**Authors:** Sajjad Ahmadi, Elaheh Haghgoshayie, Allahveirdy Arjmand, Sakineh Hajebrahimi, Edris Hasanpoor

**Affiliations:** 1 Emergency Medicine Research Team, Faculty of Medicine, Tabriz University of Medical Sciences, Tabriz, Iran; 2 Department of Healthcare Management, Research Center for Evidence-Based Health Management, Maragheh University of Medical Sciences, Maragheh, Iran; 3 Research Centre for Evidence-Based Medicine, Iranian EBM Centre: A Joanna Briggs Institute (JBI) Center of Excellence, Tabriz University of Medical Sciences, Tabriz, Iran; 4 Department of Anesthesiology, Maragheh University of Medical Sciences, Maragheh, Iran; The University of Mississippi Medical Center, UNITED STATES

## Abstract

**Background:**

Patient engagement in patient safety is aimed at increasing the awareness and participation of patients in error-prevention strategies. The aim of this project was to improve the patient safety with the patient engagement within the local context of a maternity hospital by implementing best practice.

**Methods:**

A clinical audit was conducted using the JBI Practical Application of Clinical Evidence System tool. The current project was conducted in surgical ward of Shahid-Beheshti maternity hospital, Iran. The sample size was 46 patients and 46 healthcare practitioners for both the baseline and follow-up. In phase 1, four audit criteria were used and a baseline audit was conducted for this project. In phase 2, barriers to compliance were identified, and strategies were adopted to promote best practice. In phase 3, a follow-up audit was conducted.

**Results:**

The results showed varying levels of compliance with the four criteria used in this project. The criterion 1, which was related to training of healthcare practitioners on how they can support patients, has the highest compliance at 87% in baseline and follow up data collection. Furthermore, compared with the baseline data (criterion 2 = 52%; criterion 3 = 37%; criterion 4 = 61%), compliance with criteria 2, 3, and 4 notably improved at 85, 76, and 92%, respectively.

**Conclusions:**

The present project successfully implements patient engagement in Iran and reveals varying results on compliance and the increasing knowledge of healthcare practitioners and patients on evidence-based patient engagement in order to improve the patient safety. The used strategies can facilitate implementation of evidence based procedures in clinical practice.

## Introduction

Patient safety is the prevention of errors and adverse events associated with provision of medical care [1]. Patient safety and error reduction are the shared responsibility of all healthcare professionals, and improvement depends on recruitment, education, and performance of the whole multidisciplinary team [2, 3]. A significant interest has been started internationally in involving patients in healthcare planning and service development [3, 4].

The evidence has shown that significant numbers of harmful incidents, many of which are preventable, occur in hospitals [5]. Patients are an important source of information on potentially avoidable events, and their involvement can decrease medical risks and improve outcomes [6, 7]. In general, patients agree that they should take an active role in preventing healthcare-related errors and are willing to engage with healthcare practitioners in safety practices [8]. Various strategies have been proposed to facilitate patient and/or family engagement in patient safety initiatives [8, 9].

Patient engagement in patient safety is aimed at increasing the awareness and participation of patients in error-prevention strategies. Patient engagement in healthcare planning, service development and research is a key policy component in many countries [10]. Patients are dependent on healthcare practitioners, and their decision-making [10], however, their involvement in safety initiatives is crucial to the management of long-term conditions and improving safety [4, 11]. Research has shown that, on average, patients are harmed in 10% of all hospital admissions and it is estimated that up to 75% of these incidents are preventable [1].

However, whilst the concept of “patient engagement” is recognized, unanimous definition of patient engagement exists, rather a variety of terms, including “patient involvement,” “patient collaboration,” “patient empowerment,” “partnership” and “patient-centred care,” have been used to describe a partnership with patients [12, 13].

The WHO Eastern Mediterranean Regional Office (EMRO) developed the patient safety friendly hospital initiative (PSFHI) in 2007. At first, six countries were chosen as candidate to perform the programme and later it was implemented in all other countries in the region [14]. Iran, as one of the countries of the region, participated in the programme. In the first step, 10 hospitals from around the country were selected as pilot phase. Then and according to achievements, the Ministry of Health and Medical Education (MoHME) ordered it to be implemented in about 100 hospitals in the country [15].

Patients who were aware of the need for their engagement and knowledgeable about patient safety were likely to engage in patient safety initiatives [16]. Provision of information and receiving education on how to detect and report changes in their clinical condition, and communicate errors, and how they can engage in safety initiatives can improve their engagement [9, 17]. Patients’ health status can also influence their engagement; if unable to participate, patients’ relatives may be asked to fulfil this role [4, 16]. Healthcare practitioners’ attitudes, encouragement, support and education about patient engagement in safety were identified as key to facilitate patient engagement in their safety. Healthcare practitioners also require education on how they can support patients to actively engage and how to communicate errors to each other appropriately and respectfully. Patient engagement is affected by healthcare practitioners’ knowledge, skills, and attitudes toward patient engagement and the care environment [18, 19].

Whilst research shows that patients are willing and capable of engaging in patient safety initiatives [6, 20], there remains an ambiguity over how they can become engaged in patient safety activities [21, 22], and whilst evidence of patient engagement in other aspects of health care has been well‐documented, as regards patient safety, engagement remains an emerging field of interest with limited evidence [6, 9].

Numerous studies have been conducted on the effect of patient engagement in improving patient safety. In the majority of studies, research evidence was produced and translated, but the present study tried to implement the translated evidence [4, 6, 8, 11, 13, 18, 20, 22]. Also, the aim of most previous studies was to determine the factors affecting patient engagement and patient safety [4–6, 8–18]. On the other hand, qualitative studies were conducted to explain the experiences of patients and clinicians regarding patient engagement in patient safety [3, 8, 15, 31]. So, there have been few interventional studies and clinical audit to implement the best research evidence for patient safety improvement with the patient engagement. However, this study is a clinical audit to change the behavior of clinicians and patients in the hospital. This study used the most important research evidence to be successful in implementing the interventions.

### Objective(s)

The aim of this project was to improve the patient safety with the patient engagement within the local context of a maternity hospital at Maragheh in Iran by implementing best practice recommendations.

Through the audit process, the specific objectives of the project were as follows:

- To determine current compliance with evidence-based practice regarding patient engagement in patient safety by carrying out an initial audit.
- To identify barriers and facilitators to achieving compliance and develop strategies to address areas of non-compliance.
- To improve knowledge regarding best practice regarding patient engagement in patient safety in Shahid-Beheshti hospital.
- To implement strategies for obtaining patient engagement in patient safety in order to address non-compliance with criteria.
- To conduct a follow-up audit to determine improvements in compliance with evidence-based criteria regarding obtaining patient engagement in patient safety.

## Materials and methods

### Design

The current project is a quality improvement project using the JBI Practical Application of Clinical Evidence System (JBI PACES) and Getting Research into Practice (GRiP) audit and feedback tool. The JBI PACES and GRiP framework for promoting evidence-based practice involves three phases of activity such as (1) establishing a team for the project and undertaking a baseline audit based on the criteria informed by the evidence; (2) reflecting on the results of the baseline audit and designing and implementing strategies to address noncompliance observed in the baseline audit by following the JBI and GRiP framework; and (3) conducting a follow-up audit to assess the results of the interventions implemented to improve practice and to identify future practical issues to be addressed in subsequent audits. This project was implemented in three stages, from May 2020 to October 2020.

### Ethical considerations

This project was considered a quality improvement project within the Shahid-Beheshti hospital in Maragheh, Iran. The study was approved by ethical committee of Maragheh University of Medical Sciences (Ethical code of project: MARAGHEHPHC.REC.1398.003). An approval from the hospital ethics committee was acquired. All participants entering in this study gave an informed consent. A verbal consent form was completed by all participants.

### Phase 1: Team establishment and baseline audit

#### Establishing the project team

A project team was established to engage key stakeholders to support the work during the process. The team included the senior research fellow, gynecologist, head nurse (HN), chief executive officer (CEO), chief nursing officer (CNO), chief quality officer (CQO), public relations manager and the patient safety improvement expert of the hospital. The team identified a senior nurse, clinician and a nurse educator as additional key stakeholders to support and endorse this project. Involvement of the project team was based on their roles in support, data collection, data entry and/or participation. The members of the team were invited to participate in the project based on their positive approach and ability to influence staff and to engage patients. The team leaders highlighted the importance of the recommended practice, conducted pre- and post-implementation audits based on the timeline chart. The team members and stakeholders used formal letter, phone and social media for meetings.

#### Setting and participants

The current project was conducted in surgical ward of Shahid-Beheshti maternity hospital. This hospital is a maternity health facility with a 112-bed capacity, located in the city of Maragheh. The surgical ward has 26-beds and receives approximately 2400 patients annually. The sample size included all healthcare practitioners working on this unit. There were 46 healthcare practitioners and 46 patients involved in the baseline audit, with a similar number involved in the follow-up audit.

#### Audit criteria

Table 1 shows the evidence-based audit criteria used in the project (baseline and follow-up audit) as well as a description of the sample and approaches to measure compliance with the best practice for each audit criterion. The audit criteria were translated prior to the data collection. A certified translator and one of the research team members independently translated the audit from into Persian following a forward translation method.

**Table 1:** Audit criteria, sample and approach to the measurement of compliance with best practice.

Audit criterion	Sample	Method used to measure percentage compliance with best practice
1. Healthcare practitioners have received education on how they can support patients to actively engage in patient safety practices	46 healthcare practitioners	By conducting a documentation audit, face-to-face interview, by conducting a patient survey
2. Patients have received information and education on how to detect and report changes in their clinical condition, communicate errors, and how they can best participate in patient safety initiatives.	46 patients	By conducting a patient survey
3. Where possible, patients have received specific instructions from their healthcare practitioner to take a specific action to prevent harm/adverse events or improve safety.	46 patients	By conducting a documentation audit, face-to-face interview, by conducting a patient survey
4. Visual aids such as brochures and prompts have been made available in the wards to remind patients and healthcare practitioners to perform safety behaviors.	46 healthcare practitioners 46 patients	By checking the documentations and facilities, by conducting a patient survey

#### Baseline audit

The baseline audit was conducted from May 03 to 21, 2020. The baseline audit was conducted by the project team members using the JBI PACES program. To collect the baseline data; we designed a questionnaire, consisted of demographic characteristics of the respondents (healthcare practitioners and patients) and audit criteria. Methods used to measure percentage compliance with best practice included documentation audit and semi-structured interview. The interviewers included three researchers from the research team. The researchers have previous experience conducting semi-structured interviews of patients and healthcare professionals. Additionally, they had been trained to conduct the semi-structured interviews for improving the quality of interviews. After the project team completed the audit form by reviewing of documents and interviews with participants, the investigator reviewed the participant’s chart according to criteria.

### Phase 2: Design and implementation of strategies to improve practice (GRiP)

This phase of the study focused on gaining an understanding of the barriers or gaps between the current practice and best practice in patient engagement in order to improve the patient safety. In first, the team presented baseline audit results to the healthcare practitioners. Based on these results, the team and the surgical ward identified barriers to the low compliance of the identified criteria. The team encouraged the healthcare practitioners to ask questions and suggest strategies to improve the audit results. In addition, the JBI GRiP tool was used and strategies and resources were formulated to facilitate our discussion. Then, a GRiP report was generated by outlining the implementation plan on patient engagement, and each member of surgical ward was informed.

### Phase 3: Follow-up audit post implementation of change strategy

This phase assessed whether the post-implementation resulted in the improvement of compliance with the best practice patient engagement that must be enhanced. The follow-up audit used the same criteria utilized to the baseline audit. A total of 46 patients and 46 healthcare practitioners were audited during this phase. The follow-up data were analyzed into the PACES. Results were subsequently compared with the baseline audit to determine any change in the compliance rate. This follow-up audit was conducted in late October 2020.

## Results

### Phase 1: Baseline audit

**Fig 1:**
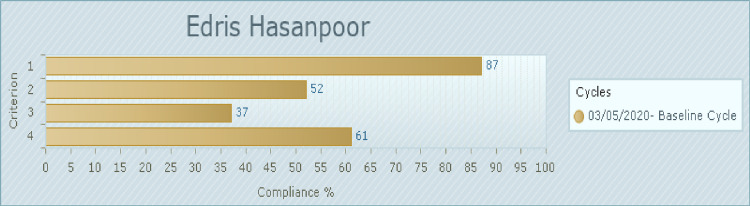
Compliance with best practice audit criteria in baseline audit (%). 1. Healthcare practitioners have received education on how they can support patients (and/or their families) to actively engage in patient safety practices. (46 of 46 samples taken). 2. Patients (and/or their families) have received information and education on how to detect and report changes in their clinical condition, communicate errors, and how they can best participate in patient safety initiatives. (46 of 46 samples taken). 3. Where possible, patients (and/or their families) have received specific instructions from their healthcare practitioner to take a specific action to prevent harm/adverse events or improve safety. (46 of 46 samples taken). 4. Visual aids such as brochures and prompts have been made available in the wards (or within the healthcare organisation) to remind patients and healthcare practitioners to perform safety behaviors. (92 of 92 samples taken).

Fig 1 indicates the baseline compliance with each audit criterion. The audit baseline results show that the healthcare practitioners have received education on how they can support patients to actively engage in patient safety practices at 87% compliance (Criterion 1). Patients (and/or their families) have received information and education on how to detect and report changes 52% compliance (Criterion 2). Compliance of the third criterion was 37% (patients have received specific instructions from their healthcare practitioner to take a specific action to prevent harm). Visual aids have been made available in the wards to remind patients and healthcare practitioners to perform safety behaviors in 61% compliance (Criterion 4).

### Phase 2: Strategies for Getting Research into Practice (GRiP)

The ward staff identified four main barriers through interviewing the participants (healthcare practitioners and patients) and then specified strategies to improve their outcomes. The project team entered the specified barriers into GRiP. The barriers and developed strategies are shown in Table 2, including resources to implement best practice. Based on the meeting and discussion with the healthcare practitioners, the head doctor of the surgical unit, and the project team, we found that providing the virtual education among healthcare practitioners and implementing the patient safety standards of hospital are the most important strategies to enhance compliance with patient engagement in patient safety. Additionally, we expressed the importance of patient involvement in improving safety for doctors by webinars and involved them in safety research. The webinars were designed with the topic “the importance of patient involvement in improving safety”. We held the webinars using the Adobe Connect and Skyroom on March 21 and 29, 2020. A total of 92 participants (46 patients and 46 healthcare practitioners) attended the webinar in two sessions, separately. Each webinar was approximately 75 minutes in length.

Subsequently, patients were encouraged to engage and communicate with their health care team. Other strategies were getting feedback from patient, attend to patients’ complaints, using the suggestion box and implementing specific guideline.

**Table 2:** Getting research into practice matrix.

No.	Barriers	Strategies	Resources	Outcomes
1	Low level of health literacy and insufficient training in patient participation	• Patient empowerment through training about medications	• Face to face education • Social networks • Pamphlet	• Increasing knowledge and Adherence to treatment and safety guides • Improvement of patients’ capacities for taking responsibility in safety practices
2	Negative attitudes toward patient involvement	• Express the importance of patient involvement in improving safety • Involvement in safety research • Encourage patients to engage and communicate with their health care team	• Journal club • Text message • Poster • Webinar	Creating positive attitude towards patient participation by improving knowledge/skills in preventing potential safety errors
3	Poor interaction between healthcare practitioners and patients	• Getting feedback from patient • Attend to patients’ complaints • Using the suggestion box • Specific guideline	• Journal club • Webinar • Suggestion box • Booklets	• Improving the quality medical communications in the unit
4	Workload	• Employ more nurses in surgical ward and reduce workload • Incentive mechanisms	• Nurses as educators • Financial resources	• Professional/work satisfaction

### Phase 3: Post-implementation audit

**Fig 2:**
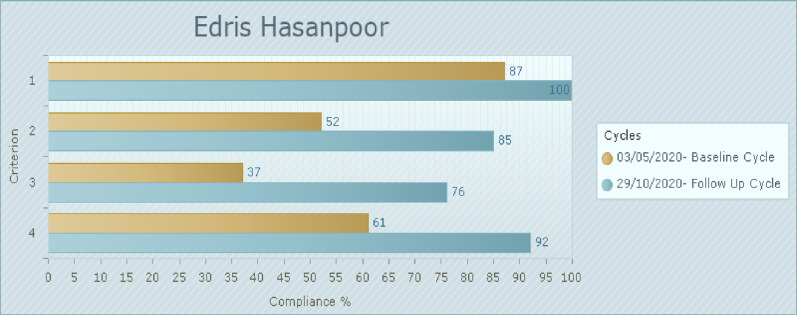
Compliance with best practice criteria in follow-up audit compared to baseline audit (%). 1. Healthcare practitioners have received education on how they can support patients (and/or their families) to actively engage in patient safety practices. (46 of 46 samples taken). 2. Patients (and/or their families) have received information and education on how to detect and report changes in their clinical condition, communicate errors, and how they can best participate in patient safety initiatives. (46 of 46 samples taken). 3. Where possible, patients (and/or their families) have received specific instructions from their healthcare practitioner to take a specific action to prevent harm/adverse events or improve safety. (46 of 46 samples taken). 4. Visual aids such as brochures and prompts have been made available in the wards (or within the healthcare organisation) to remind patients and healthcare practitioners to perform safety behaviors. (92 of 92 samples taken).

Fig 2 reflects the baseline and the follow-up audit and compliance report according to each audit criterion involving 46 participants. Criterion 1 (training of healthcare practitioners on how they can support patients) remained at 100% compliance. Furthermore, compared with the baseline data, compliance with criteria 2, 3, and 4 notably improved at 85, 76, and 92%, respectively.

## Discussion

This project was the first attempt to examine the current practice and implement evidence-based patient engagement in patient safety in a maternity hospital in Maragheh, Iran. Baseline and follow-up data were collected, and barriers, strategies, and resources were identified using the JBI PACES and GRiP tools. The project team and hospital leadership provided the resources in order to implement the identified strategies. The importance and practices of facilitators of patient engagement in patient safety were included in the educational programs of healthcare practitioners in the surgical ward. In addition, educational content were provided in unit and meetings were conducted to review patient engagement in patient safety.

The results showed that after implementation of the strategies, highest mean scores of criterion were found in relation to training of healthcare practitioners on how they can support patients (100%). The compliance rate for the criterion 2 on patients have received information and education on how to detect and report changes in their clinical condition, communicate errors, and how they can best participate in patient safety initiatives increased from 52% to 85%. The compliance rate of criterion 3 in relation to patients have received specific instructions from their healthcare practitioners to improve safety increased from 37% to 76% in the post implementation period. Finally, the compliance rate for the criterion 4 on preparation of the visual aids such as brochures to remind patients and healthcare practitioners to perform safety behaviors increased from 61% to 92%.

Despite the successful implementation of the project, several limitations exist. The first was inability to implement the face-to-face training to healthcare practitioners because of the outbreak of coronavirus disease (COVID-19). The project was conducted during this outbreak, which is a less busy time for healthcare practitioners. Therefore, the workload has increased that can influence the strategies. Additionally, due to the time limitations and funding, the strategies were only implemented in one ward and in one hospital. Therefore, the sample size was small and composed patients and healthcare practitioners from one maternity hospital so may not be generalisable to other hospitals.

This project has several strengths. One of the strengths of this study is to implement the best translated research evidence related to patient safety improvement using the patient engagement. We tried to change the behaviour of healthcare practitioners and patients in order to improve the patient safety and engagement in hospital. Also, our method of study was unique compared to other previous studies. We established a project team to support the work during clinical audit process in medical practice. The team included the key stakeholders. Then, the evidence-based audit criteria used in the project (baseline and follow-up audit). Next, we implemented evidence-based strategies to improve performance using JBI GRiP tool. Finally, we assessed post-implementation resulted in the improvement of compliance with the best practice patient engagement. In fact, we modified and improved the performance of patients and healthcare practitioners using the best available research evidence.

A challenge for the implementation of the project was that some of the healthcare practitioners did not think it was necessary to conduct the assessment, because patient safety standards are implemented in the hospital. In order to overcome the challenge and improve the beliefs of healthcare practitioners of the importance of conducting a project, a webinar about patient engagement in patient safety and practice improvement of clinicians were conducted and then, strategies were provided by considering their perspectives.

The main success of the project was including the full support of the hospital management team. Hospital leadership has important responsibility to oversee the safety and quality of care provided [23]. The studies showed that leadership support for patient safety is of particular importance in small hospitals where the economic burden of safety programs is disproportionately great and leadership is closer to the frontlines [23, 24]. The second success of this project was good performance of nursing team in patient safety programs related to hospital accreditation standards [25, 26]. In addition to these two, the implementations of developed strategies lead to synergy effects in the audit process.

In this project, barriers to the engagement of patients in the delivery of safe care in Shahid-Beheshti hospital were identified. The results highlighted that low level of health literacy and insufficient training in patient participation are disproportionate to the number of patients and that this decreases patients’ ability to take an active role in safety. A systematic review also showed the insufficiency of health literacy amongst the Iranian population [27]. In order to overcome the barrier, we implemented the strategy of patient empowerment through training about patient safety. One of the most important barriers to the engagement of patients is negative attitudes toward patient engagement. In relation to the strategies used to implement best practice in patient engagement, the use of a journal club, poster and webinar to supplement healthcare practitioners’ education about importance of patient involvement in improving safety. Additionally, healthcare practitioners linked to the clinical research development unit (CRDU) of hospital in order to involve in safety researches. Patients were encouraged by text messages to engage and communicate with their health care team. Forbat and colleagues found that direct experience of participatory working is to lead to positive attitude in the perspectives of healthcare practitioners [28]. Another barrier to the patient engagement was poor interaction between healthcare practitioners and patients. We tried to overcome this barrier by using the specific guidelines regarding the patient safety for healthcare practitioners and the suggestion box for investigating the patient perspectives. The results indicated that relationship skills can lead to efficiency, safety, and clinical outcomes [29–31]. Finally, the participants believed that the workload of healthcare practitioners was the principle barrier for effective patient‐provider relationship and patient engagement. We used the strategies of incentive mechanisms and employing more nurses to reduce workload in unit. The evidence showed that workload in Iranian hospitals is to an important barrier in effective patient engagement [32, 33].

## Conclusions

This project was successful to an important evidence for improving healthcare practitioners’ skill and knowledge about evidence-based patient engagement in patient safety in Shahid-Beheshti hospital. The results of this project provide the positive direction into implementing evidence-based patient engagement in other hospitals. This project has the potential to raise awareness amongst healthcare practitioners and managers on the barriers to patient engagement in patient safety and improvement strategies in Iranian hospitals.

In the future, follow-up audits engaging other clinicians from the hospital units should be conducted. Thus, healthcare practitioners and patients will be empowered to improve performance and patient safety. This project is a critical point in patient safety improvement with the patient engagement in Maragheh. We must try to provide the formal educational and practical strategies for sustainability.
